# Implication of Long noncoding RNAs in the endothelial cell response to hypoxia revealed by RNA-sequencing

**DOI:** 10.1038/srep24141

**Published:** 2016-04-11

**Authors:** C. Voellenkle, J. M. Garcia-Manteiga, S. Pedrotti, A. Perfetti, I. De Toma, D. Da Silva, B. Maimone, S. Greco, P. Fasanaro, P. Creo, G. Zaccagnini, C. Gaetano, F. Martelli

**Affiliations:** 1 Laboratory of Molecular Cardiology, Policlinico San Donato-IRCCS, 20097 Milan, Italy; 2 Center for Translational Genomics and Bioinformatics, San Raffaele Scientific Institute, 20132 Milan, Italy; 3 Laboratory of Stem Cells for Tissue Engineering, Policlinico San Donato-IRCCS, 20097 Milan, Italy; 4 Division of Cardiovascular Epigenetics, Goethe University, 60590 Frankfurt am Main, Germany

## Abstract

Long noncoding RNAs (lncRNAs) are non-protein coding RNAs regulating gene expression. Although for some lncRNAs a relevant role in hypoxic endothelium has been shown, the regulation and function of lncRNAs is still largely unknown in the vascular physio-pathology. Taking advantage of next-generation sequencing techniques, transcriptomic changes induced by endothelial cell exposure to hypoxia were investigated. Paired-end sequencing of polyadenylated RNA derived from human umbilical vein endothelial cells (HUVECs) exposed to 1% O_2_ or normoxia was performed. Bioinformatics analysis identified ≈2000 differentially expressed genes, including 122 lncRNAs. Extensive validation was performed by both microarray and qPCR. Among the validated lncRNAs, H19, MIR210HG, MEG9, MALAT1 and MIR22HG were also induced in a mouse model of hindlimb ischemia. To test the functional relevance of lncRNAs in endothelial cells, knockdown of H19 expression was performed. H19 inhibition decreased HUVEC growth, inducing their accumulation in G1 phase of the cell cycle; accordingly, p21 (CDKN1A) expression was increased. Additionally, H19 knockdown also diminished HUVEC ability to form capillary like structures when plated on matrigel. In conclusion, a high-confidence signature of lncRNAs modulated by hypoxia in HUVEC was identified and a significant impact of H19 lncRNA was shown.

The response of mammalian cells to internal and external stimuli requires the accurate modulation of gene expression patterns, regulated at the transcriptional and post-transcriptional level. Transcriptional control occurs mainly by changes in chromatin composition and in transcription factor recruitment. Post-transcriptional regulation can be mediated by differential splicing of pre-mRNAs and alterations in the stability, transport and translation of mRNAs^1^. Noncoding RNAs influence each of these gene regulatory levels, and among these, long noncoding RNAs (lncRNAs) represent the most prevalent and functionally diverse class^2^. LncRNA genes are transcribed by RNA polymerase II, exist as single or multi-exonic transcripts, undergo 5’capping and splicing, and can be either polyadenylated or not^3^. In general, lncRNAs display poor conservation and low expression levels^3^. LncRNAs can be classified according to their genomic location and context: lincRNAs are expressed from intergenic regions, antisense lncRNAs are transcribed from the opposite strand of a protein-coding mRNA, sense intronic ncRNAs are present in introns of annotated genes, and sense lncRNA overlap also with exons from protein-coding gene^4^. For this heterogeneous class of ncRNAs, various molecular mechanisms for the regulation of gene expression have been identified. Some nuclear lncRNAs regulate the state of the chromatin (epigenetic regulation), while other lncRNAs modulate transcription initiation and elongation (transcriptional regulation)^5^. lncRNAs can also act as endogenous sponges for other RNAs such as mRNAs and miRNAs, as molecular guides and scaffolds to interact with protein complexes or as molecular decoys for proteins, including transcription factors (post-translational regulation)^6^. LncRNA genes can exert important functions even at low copy number^7^ and their regulation can be triggered by external stimuli, including low oxygen (O_2_) tension^8^.

O_2_ is critical to survival due to its function as the final electron acceptor in the mitochondrial respiratory chain. Thus, O_2_ deficiency may lead to the death of cells, tissue, or even the whole organism. All cells have the ability to sense O_2_ concentration and to respond to reduced O_2_ availability (hypoxia). Hypoxia can occur during embryonic development and pathophysiological events, like myocardial infarction, peripheral ischemia or stroke, triggering a distinct program of responses aimed to relieving tissue hypoxia and removing irreversibly damaged cells. The adaptive response to hypoxia mediated by the vasculature include EC proliferation, migration and angiogenesis, as well as growth arrest and apoptotic cell death. The master regulator of O_2_ homeostasis is Hypoxia-Inducible Factor 1 (HIF-1), a transcriptional activator orchestrating a multitude of biological processes and controlling the delivery and use of O_2_. HIF-1 plays a critical role in angiogenesis, mediating the vascular responses to hypoxia and ischemia^9^.

Several reports have shown that certain lncRNAs play a significant role in the hypoxic response of ECs. The first lncRNA to be described in the endothelium was an antisense transcript of the EC-restricted NOS3 gene (noncoding isoform of ATG9B). The expression of this lncRNA in ECs is necessary and sufficient to attenuate the levels of NOS3 protein with little effect on the mRNA levels^10^. RNA-sequencing analysis of HUVECs revealed high expression levels of MALAT1, a conserved lincRNA induced by hypoxia. Knockdown of MALAT1 in ECs results in a pro-migratory phenotype *in vitro* and reduced capillary density and blood flow in a hindlimb ischemia model^11^. Recently, hypoxia-sensitive lncRNAs in HUVEC were investigated by RNA-sequencing and microarray approaches. Two of the identified lncRNAs, LINC00323-003 and MIR503HG, were further characterized by applying loss- and gain-of-function approaches, revealing an important role for both of these lncRNAs in endothelial biology^12^.

Although the converging fields of bioinformatics, genomics, and next generation sequencing have revealed the importance of several lncRNAs in EC, lncRNA regulation and role in the vasculature is largely unknown^3^,^13^,^14^.

In this study, the power of next-generation sequencing technology was employed to delineate the coding and non-coding transcriptome of human EC in great depth and to identify the modulations induced by hypoxia.

## Results

### RNA library generation, sequencing and composition

**Figure 1:**
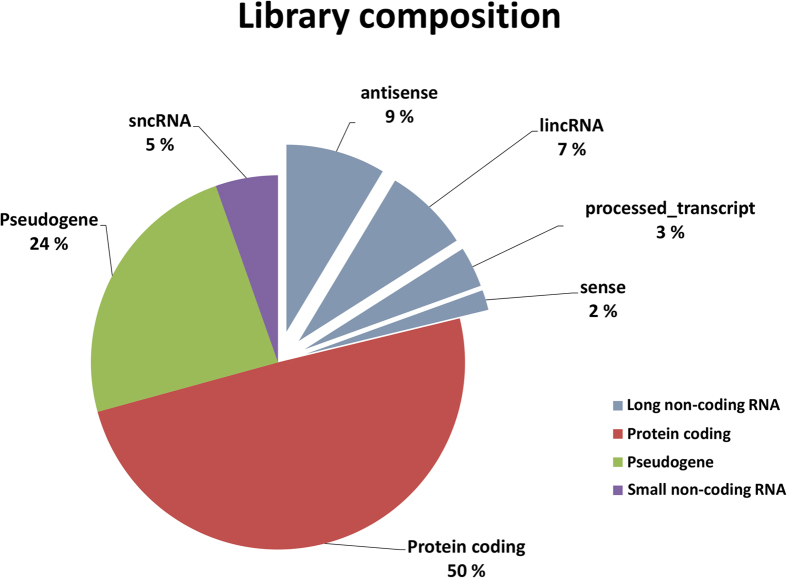
Library composition. Pie chart of the distribution of detected genes into four summarized transcript types as indicated by Ensembl 72, expressed in percentages. LncRNAs are further divided into the following classes: antisense, long intergenic ncRNAs (lincRNAs), processed transcripts, sense-intronic and sense-overlapping (sense).

A total of 8 barcoded cDNA libraries were prepared from HUVEC cells exposed to 24 hours normoxia or to hypoxia for 24 and 48 hours. Altogether, ≈1,300 million paired-end reads were generated by next-generation sequencing and filters for quality and unique alignment to the human genome were applied. Among the remaining 1,200 million forward and reverse reads, 93% were found to be properly paired. Of the fragments constituted by correctly paired reads, 500 million aligned unambiguously to exons annotated in Ensembl (GRCh37/hg19, Feb 2009, Version 72) (Supplementary Table S1). Among the ≈35,000 genes detected, 50% represented protein-coding transcripts, 21% were assigned to long and 5% to short non-coding transcript types, 24% were identified as pseudo-genes (Fig. 1 and Supplementary Fig. S1). Of the lncRNAs, 9% were annotated as antisense and 7% as long intergenic RNAs, 3% belonged to processed-transcripts and 2% were sense-intronic or sense-overlapping RNAs (Fig. 1). Distribution of the average expression per gene between the gene classes showed that 85% of the raw counts were produced by protein-coding genes, while only 2% resulted from lncRNAs (Supplementary Fig. S1).

### Differential Expression, Validation and Enrichment Analysis of the Genes

The expression levels of the identified transcripts were quantified, a filter for abundancy was applied and differences in expression between normoxia and hypoxia were determined (Supplementary Table S2). The datasets of 24 and 48 hours hypoxia HUVECs were analyzed together and compared to the normoxia datasets in order to increase the statistical power of the study and to concentrate on transcripts that were robustly modulated by hypoxia over time. This analysis yielded ≈2,000 significantly differentially expressed genes (Supplementary Table S2).

**Figure 2:**
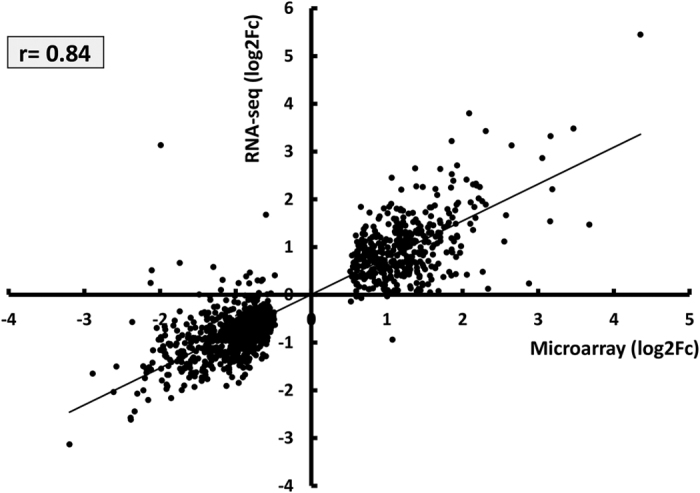
Correlation of HUVEC profiles obtained by RNA-sequencing and Microarrays. Expression profiles of hypoxic HUVEC (n = 4) obtained by next-generation sequencing were compared to profiles obtained by microarray of an independent set of hypoxic HUVEC, constituted by normoxia (n = 2), 24 h hypoxia (n = 2) and 48 h hypoxia (n = 2) cells. The graph shows the Spearman correlation of the two datasets of differentially expressed transcripts (alpha = 0.05; two-tailed p < 0.0001).

For validation purposes, the hypoxic signature derived by RNA-sequencing was compared to gene expression profiles obtained by microarray analysis in independent experiments, revealing a highly significant correlation between the two different approaches (Fig. 2).

**Figure 3:**
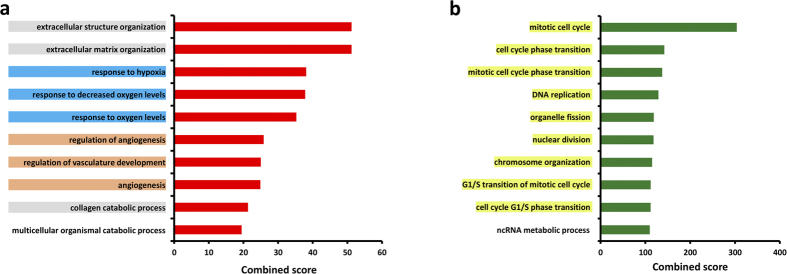
Enrichment analysis of Gene Ontology Biological Processes (GOBP) of genes modulated in HUVEC upon hypoxia exposure. Top 10 GOBP ranked by combined score associated with **(a)** up-modulated and **(b)** down-modulated signatures using Enrichr analysis tool. Terms belonging to closely related categories are highlighted in the same color. A Benjamini-Hochberg adjusted p-value < 0.05 was used as significance threshold.

In order to identify relevant biological functions, the obtained signature was separated into up-and down-modulated genes and Gene Ontology enrichment analysis of Biological Processes (GOBP) was performed. Genes induced upon hypoxia showed a significant involvement mainly with categories related to extracellular space remodeling, response to hypoxia and angiogenic processes (Fig. 3a). Whereas genes repressed upon hypoxia revealed significant enrichment among various categories of the cell cycle (Fig. 3b). Accordingly, ChIP Enrichment analysis (ChEA) suggested HIF1a and closely related co-players EP300 and GATA2 as key transcription factor for up-modulated genes (Supplementary Fig. S2a). ChEA of the down-modulated signature pointed to members of transcription factor families crucial for cell cycle control, like E2F4, E2F7^15^ and FOXM1^16^ (Supplementary Fig. S2b).

### Differentially expressed lncRNAs in hypoxic ECs

**Figure 4:**
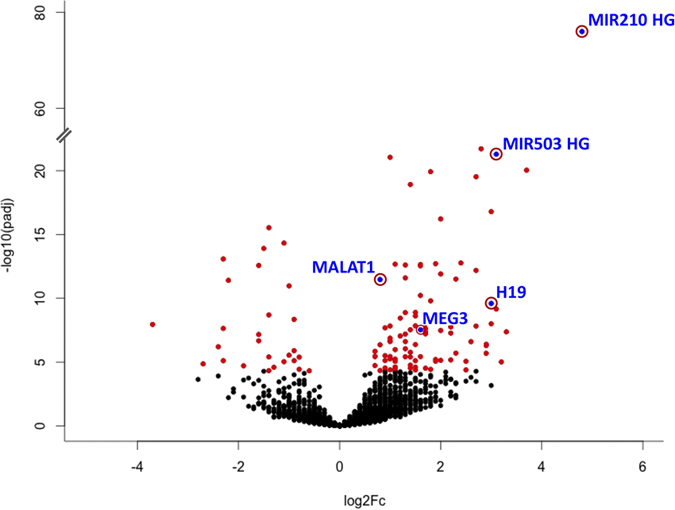
Volcano plot of differentially expressed lncRNAs in hypoxic HUVEC. 122 lncRNAs were identified as significantly modulated upon hypoxia, applying adjusted p-value < 5 × 10^−5^ as threshold (red dots). LncRNAs of particular interest are displayed as blue dots circled in red.

Among all detected genes, ≈7,400 transcripts are annotated as long-non coding RNAs (Supplementary Table S2). Within these, 122 lncRNAs were found to be differentially expressed (Fig. 4 and Supplementary Table S3). The lncRNA species with the highest and most significant modulation was MIR210HG, the precursor transcript of miRNA-210, a known master-hypoxamiR induced upon hypoxia^17^. Interestingly, several imprinted maternally expressed lncRNAs were found to be induced upon hypoxia as well, like MEG3, MEG8, MEG9 and H19 (Table 1).

In order to validate the hypoxic signature, a subset of candidates was tested by qPCR, including also several lncRNAs of special interest above the significance threshold. A variety of criteria was used to select the lncRNAs for validation: lncRNAs already described in other systems to be modulated upon hypoxia; lncRNAs conserved in mouse; lncRNAs antisense of a protein-coding gene; imprinted lncRNA. Out of 24 candidates, 20 lncRNA species confirmed the significant modulation identified by software analysis, divided in 16 up- and 4 down-modulations (Table 1). Notably, 4 of these validated lncRNAs were candidates showing an adjusted p-value above the threshold of 5 × 10^−5^ (Table 1).

Out of the 122 modulated lncRNAs, 30 belong to the antisense class. For each of these, we screened for “near protein-coding genes” on the opposite strand and filtered for significance. In this way, we identified 8 significantly modulated protein coding genes (Supplementary Table S4). Three potentially interesting antisense lncRNAs not present in the signature, but validated by qPCR, were included. For those, two nearby protein-coding sense transcripts revealed significant differential expression. (Supplementary Table S4). Interestingly, Enrichr bioinformatics analysis showed a significant relation to two NCI-nature pathways: “HIF-2-alpha transcription factor network” and “BARD1 signaling events ” (data not shown).

### Hypoxia induced lncRNAs are increased upon ischemia

To validate the induction by hypoxia of the identified lncRNAs *in vivo*, we took advantage of a mouse model of hindlimb ischemia. In this model, the femoral artery is dissected, inducing acute ischemia in the lower leg muscles^18^.

**Figure 5:**
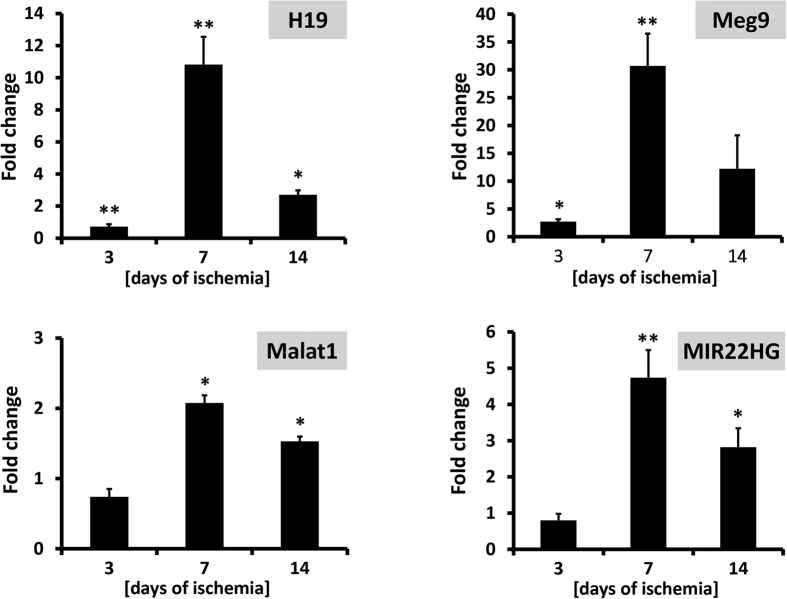
Conserved lncRNAs validated in mouse hindlimb ischemia. Unilateral hindlimb ischemia was induced by femoral artery dissection. Bar graphs show the modulation of the indicated lncRNA in ischemic gastrocnemius muscles compared with non-ischemic controlaterals (n = 3; *p < 0.05, **p < 0.01).

The lncRNAs confirmed by qPCR were screened for mouse homologues, identifying 6 conserved lncRNAs: H19, EGOT, MALAT1, MEG8_1, Mir22hg and Mirg-010 (=Meg9). Each of them was checked for deregulation in ischemic gastrocnemius muscles, at 3, 7 and 14 days after surgery. Indeed, for H19, Meg9, MIR22HG and Malat1 significant up-modulation was found, with an induction maximum at 7 days of ischemia (Fig. 5).

A mouse homologue of human MIR210HG, the most induced lncRNA in hypoxic HUVEC, has not been identified yet. In an effort to identify the host gene of mouse miR-210, Cufflinks suite was used to analyse publicly available RNA-sequencing data derived from hypoxic 3T3-L1 mouse cell line^19^. By this means, a candidate transcript in the locus of miR-210 was identified, showing increased expression levels in low-oxygen conditions (Supplementary Fig. S3a). Hypoxic NIH3T3 fibroblasts were then used to further investigate this candidate transcript by PCR and Sanger sequencing. Indeed, the expression of 5 fragments within the proposed transcripts borders could be confirmed (Supplementary Fig. S3a). Four of these fragments showed significant differential expression upon hypoxia (Supplementary Fig. S3b). The fragment containing miR-210 displayed low expression levels (raw Ct>33), therefore conclusive results about its induction were not possible.

Next, fragments 3 and 5 were tested in ischemic muscles to assess the induction of the potential host gene of miR-210 *in vivo*. Supplementary Fig. S3c shows that indeed both fragments were induced in ischemic gastrocnemius muscles at day 7 after surgery. While a complete characterization of the host gene of miR-210 in mouse is still required, we can conclude that the potential precursor of miR-210 is induced by hypoxia *in vivo*.

### HIF1 occupancy and Hypoxia Responsive Elements (HRE) presence in the hypoxic lncRNA signature

In order to identify HIF1 bindings sites in the regions of the lncRNAs comprised in the hypoxic signature, we took advantage of publicly available ChIP-seq data for HIF1-alpha, HIF1-beta, and HIF2-beta in hypoxic breast cancer MCF-7 cells^20^. Out of 122 hypoxia-induced lncRNA genes, 51 had at least one significant peak for one or more “HIF1” proteins in the region covering the whole gene body and the predicted promoter (5 kbp upstream the transcription start site). Intriguingly, 80% of these lncRNAs showed upregulation upon hypoxia and 26 featured at least one HRE with a minimum conservation score of 80% (Supplementary Table S5). Noteworthy, 10 of the validated lncRNAs showed at least one significant peak for “HIF1” proteins and 6 of these, comprised at least one HRE with a minimum conservation score of 80% (Supplementary Table S5). Among the validated lncRNAs, the highest number of HREs was identified for MIR210HG, where 4 HREs were found in the region immediately upstream of the transcript (Supplementary Fig. S4).

### Vascular expression of H19 *in vivo*

Given that H19 is expressed during the development of rat aorta, decreases in adults, and, interestingly, increases after vascular injury^21^,^22^, we focused on H19 function in EC. First, to establish if any impact of H19 observed in cultured EC could be relevant for the endothelium in a tissue context, the expression of H19 RNA was investigated by *in situ* hybridization (ISH) in frozen human muscle sections. We found that H19 RNA was mostly expressed in vascular tissues (Supplementary Fig. S5a–c). The vascular localization of H19 RNA was confirmed by immunohistochemistry for CD31 on serial sections (Supplementary Fig. S5d).

### H19 blocking influences EC transcriptome, induces EC growth arrest and impairs capillary-like formation

The effect of H19 expression levels on cellular processes in EC was investigated measuring the impact on the transcriptome of H19 inhibition. To this aim, H19 was knocked down by transfection of an antisense oligonucleotide (LNA GapmeR) in human aortic EC (HAOEC; Supplementary Fig. S6a). Gene expression levels were then estimated by microarray-analysis, detecting ≈12,900 out of a total of ≈31,400 genes present on the chip. Differential expression analysis resulted in a signature of 351 genes (Supplementary Table S6).

Of relevance, WebGestalt (WEB-based GEne SeT AnaLysis Toolkit)^23^ identified a significant enrichment of signature-genes mainly related to vascular and cardiovascular diseases (Supplementary Fig. S7).

**Figure 6:**
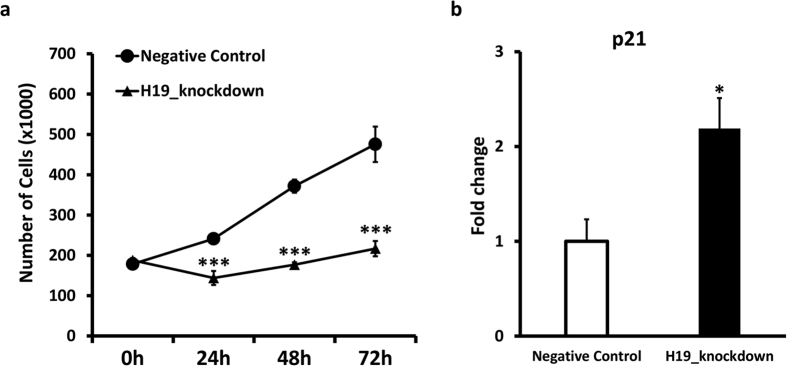
H19 knockdown leads to reduced proliferation and activates p21/CDKN1A. HUVEC were transfected with an antisense LNA GapmeRs specific for H19 or with negative control. (**a)** Seeding for proliferation assays was performed 24 h after transfection. Growth curves show a significant impact of H19 knockdown compared to control at all investigated time-points (n = 9; ***p < 0.001). (**b)** The bar graph shows increased p21/CDKN1A expression upon H19 knockdown, as measured by qPCR (n = 6; *p < 0.05).

**Figure 7:**
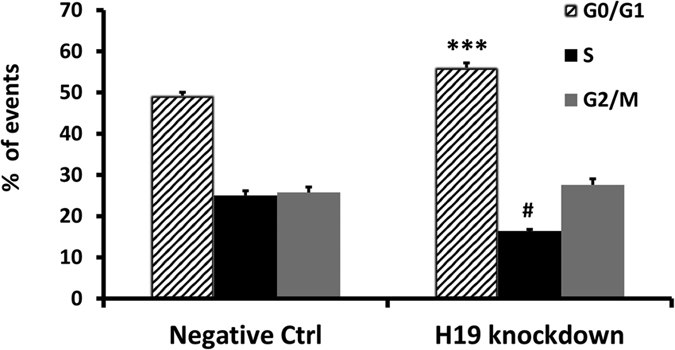
H19 knockdown affects the cell cycle by diminishing cell number in G0/G1 and S phases. 48 hours after transfection with H19 antisense oligonucleotide or negative control, HUVEC were labelled with EdU. Next, incorporated EdU was revealed with an Alexa Fluor 647-labelled antibody and total DNA content with propidium iodide. Cell cycle analysis indicated a significant reduction of cells in G0/G1 and S phases (n = 6, ***p < 0.001, ^#^p < 0.0001).

To validate experimentally the effect of H19 expression on EC function, H19 expression was knocked down by LNA GapmeR transfection (Supplementary Fig. S6b) and cell growth was monitored. Figure 6a shows that H19 inhibition induced a significant reduction of cell number compared to control, at all time points. For a better understanding of the nature of the observed impact on proliferation, the effect of H19 knockdown on cell cycle and cell death was investigated. The differential expression of cyclin/CDK inhibitors upon H19 knockdown was analysed by qPCR, revealing the upregulation of p21/CDKN1A (Fig. 6b) and p16, while p18 was down-modulated (Supplementary Fig. S8a). Remarkably, RNA-sequencing data showed that p21/CDKN1A constituted >80% of the overall expression of all 7 cyclin/CDK inhibitors analysed (Supplementary Fig. S8b). Accordingly, flow cytometry analysis of cell cycle phases revealed a significant accumulation of cells in G0/G1 phase (+8%) and a corresponding significant decrease in S-phase (−9%) upon H19 knockdown (Fig. 7 and Supplementary Fig. S8c). Additionally, apoptosis assay indicated a minimal, though statistically significant increase in apoptotic DNA fragmentation upon H19 knockdown (Supplementary Fig. S9).

**Figure 8:**
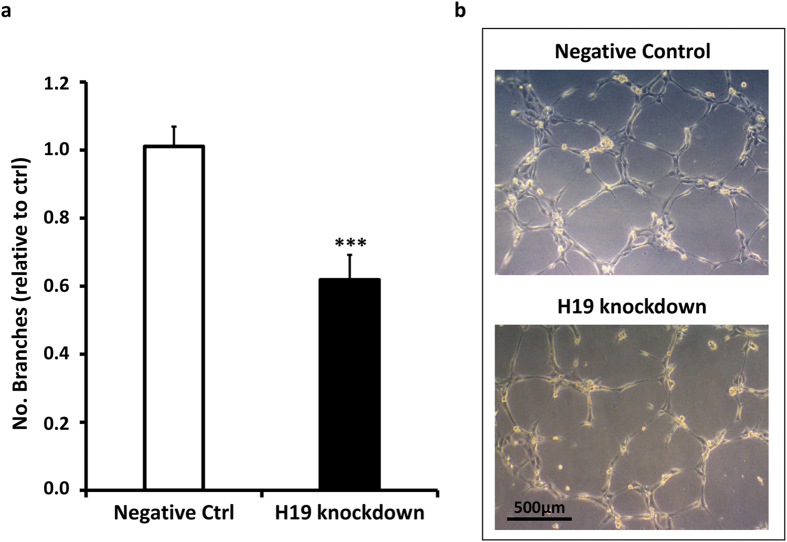
H19 knockdown decreases the formation of capillary-like structures. HUVEC were transfected with an antisense LNA GapmeRs specific for H19 or with negative control. The ability to form capillary like structures was quantified by matrigel-assay 48h after transfection. (**a**) The bar graph shows a significant reduction of the number of branches upon H19 knockdown versus control (n = 9, ***p < 0.001). (**b**) Representative phase-contrast images of the organization into capillary-like structures are shown.

One way to assay certain features of angiogenesis is to exploit the ability of EC to form capillary-like structures on Matrigel^24^. This *in vitro* approach showed that transient H19 knockdown significantly decreased the ability of HUVEC to form capillary-like structures (Fig. 8a,b).

To rule out unspecific off-target effects, cell growth measurements and matrigel-assays were repeated using a different antisense-sequence for H19 knockdown (LNA GapmeR 3) (Supplementary Fig. S6c). Indeed, both approaches confirmed the findings of the H19 knockdown experiments performed previously (Supplementary Fig. S10).

Instead, when H19 was overexpressed by lentiviral infection of HUVEC (Supplementary Fig. S6d), no significant alteration of proliferation rate or capillary-like structure formation was observed (Supplementary Fig. 11).

## Discussion

In the present study, the differential expression landscape of protein-coding and non-coding transcripts in hypoxic ECs was delineated by next-generation sequencing. We analyzed more than 100 million of paired-end reads per sample, allowing the measurement of low expressed mRNAs and lncRNAs. Validation performed both by qPCR and microarray analysis confirmed the accuracy of our analysis. Accordingly, the genome-wide distribution of the identified protein-coding mRNAs and lncRNA classes was comparable to that found by others in EC^11^.

It is worth mentioning that, for comparison between studies, it is crucial to consider the measurement technique and the annotation used for lncRNA identification. Indeed, criteria for naming, categorizing and validating lncRNAs are not well defined yet^3^. Several databases exist, containing very different numbers of lncRNA genes^3^. For instance, a very recent lncRNAs profiling performed by microarray in macrophages has suggested that ~60% of lncRNAs belong to the class of intergenic lncRNAs^25^. The apparent discrepancy with our findings (9% antisense lncRNAs and 7% intergenic) might be easily explained considering the technological and annotation differences.

One limitation of this study is that, since our experimental procedure included oligo (dT) purification of total RNA, non-polyadenylated transcripts were not detected. Although the majority of the lncRNA is polyadenylated, a significant portion of the transcripts is either poly (A)− or bimorphic^26^. Also, potential shifts in abundance between poly (A)+ and poly (A)− transcripts of bimorphic lncRNAs modulated by hypoxia, like Malat1 or NEAT1^27^, were not detected by our study.

Remarkably, the large majority of the RNA expression resulted from the protein-coding fraction, while only 2% was attributable to lncRNAs. These findings are in agreement with a recent attempt to capture the spectrum of human transcripts by analyzing the transcriptome of 27 different tissues, emphasizing the high tissue specificity and generally low expression levels of lncRNAs^28^.

The biological relevance of the obtained gene expression profiles was also confirmed by the bioinformatics enrichment analysis of both functional categories and transcription factors. Indeed, gene ontology analysis positioned not only “response to hypoxia” categories amongst the most significant, but also “extracellular space remodelling” and “angiogenic processes”, both known to be prominent features of the response of EC to hypoxia^9^,^29^,^30^. Moreover, HIF1a, the master regulator of oxygen homeostasis^29^, its co-activator Ep300^9^,^31^ and GATA2, a cooperating transcription factor for induction of HIF1a target genes^32^, were suggested as key transcription factors for the regulation of up-modulated genes. Expectedly, top ranking functional categories identified for the down-modulated signature are involved in cell cycle, its arrest being the classical cellular response to hypoxia. Furthermore, the bioinformatics analysis pointed to transcription factor families known to be key regulators of cell cycle progression, E2F^15^ and Forkhead box (Fox)^16^.

Applying a stringent significance threshold, 122 lncRNAs were identified to be differentially expressed upon low O_2_ levels, suggesting a prominent role of the lncRNAs in the EC response to hypoxia.

Intriguingly, the lncRNA species with the highest and most significant modulation was MIR210HG, the precursor of miRNA-210, identified by our group and others a master hypoxamiR^17^. In addition, MIR210HG induction upon hypoxia was observed before in hypoxic and inflammatory renal epithelial injury^33^.

Interestingly, four imprinted maternally expressed lncRNAs, i.e. MEG3, MEG8, MEG9 and H19 were found to be induced upon hypoxia. This leaves room for speculations about a possible role of hypoxia and epigenetic regulation involving lncRNA similar to the mechanism shown for the imprinted WT1 and its antisense lncRNA^34^.

In keeping with our findings, the modulation upon hypoxia of MIR503HG, linc00323^12^ Meg3 and Malat1^11^ was reported recently.

Noteworthy, for the 33 antisense lncRNAs present in the identified signature or validated by qPCR, 10 near protein-coding genes were found to be significantly modulated. Either concordant or discordant modulation can have biological relevance, since antisense lncRNAs can act both positively and negatively on their protein-coding counterpart^35^. Among these 10 coding genes, several were implicated in the context of hypoxia response.

Not surprisingly, screening of the validated lncRNAs for mouse homologues revealed only six conserved, emphasizing poor conservation of lncRNAs between species^36^. Nevertheless, for four lncRNAs the expected significant induction upon low oxygen levels could be confirmed in a mouse model of hindlimb ischemia. Intrigued by the features of human MIR210HG, we attempted to characterize its murine counterpart. A hypoxia induced transcript in the region of miR-210 was identified by computational approach using public RNA-sequencing data of low-oxygen mouse cells. Indeed, the expression of sequence fragments covering nearly the complete transcript could be confirmed in cultured mouse fibroblasts and in a mouse model of hindlimb ischemia. Although further investigations are necessary to identify the precise structure, in the present study, first evidence of the presence of mouse miR-210 host gene was provided.

HIF1 binding sites across the signature-lncRNAs were identified by taking advantage of a publicly available Chip-sequencing study on hypoxic breast cancer cells^20^. Remarkably, for 40% of the lncRNAs HIF1-occupancy was found within the promoter and gene body region, and the majority of these were induced upon hypoxia. Furthermore, 50% featured also one or more highly conserved HRE. Interestingly, four highly conserved HRE were present in the upstream region of MIR210HG, in keeping with previous promoter functional analysis^37^. These findings corroborate the indication of HIF as a major regulator of lncRNAs modulation upon hypoxia^38^. Given that the HIF occupancy data is derived from a different cell type, one might speculate that the identified lncRNAs expression pattern might be similar, at least in part, also in other systems.

Noteworthy, HREs in the regulatory region of MIR210HG have been previously validated experimentally in different systems^37^,^39^,^40^. Indeed, the HIF1 binding site validated by Huang *et al*.^39^ in squamous cell carcinoma cells is identical to the HRE#3 identified by us. Instead, there was no overlap with any of the three HRE confirmed in HT-29 cells by Kulshreshtha *et al*.^40^, likely due to differences in the experimental systems.

Previous studies showed that H19 is expressed during the development of rat aorta, decreases in adults, and increases after vascular injury^21^,^22^, indicating a pervasive role of H19 in vascular physio-pathology; thus, the function of H19 in EC was explored. We found that H19 increased in hypoxic ECs and upon limb ischemia, in agreement with data obtained in other cell types^41^. Additionally, H19 decrease had a significant impact on EC transcriptome and pathway analysis indicated H19 involvement in vascular physio-pathology.

Noteworthy, H19 can be either a tumor suppressor or a promoter of cancer metastasis by epigenetic modification functions^41^,^42^,^43^. We found that H19 inhibition resulted in a dramatic reduction of EC growth and of capillary-like structure formation. Indeed, both findings are in keeping with the pro-proliferative and pro-angiogenic roles of H19 in cancer cells^44^ and with the ability of H19-carrying exosomes derived from hepatoma cells to stimulate EC tubulogenesis^43^.

Apoptosis assay did not show a biologically significant increase of EC death, while cell cycle analysis pointed to cell cycle arrest at the G1/S boundary. These findings are in accordance with the observed modulation of the Cyclin/CDK inhibitors upon H19 knockdown. While p21/CDKN1A and p16/CDKN2A were upregulated, p18/CDKN2C showed a similarly strong repression. Given that Cyclin/CDK inhibitors function as a pool^45^, their relative levels of gene expression is of importance. As identified by RNA-sequencing, p21/CDKN1A shows >80% while p16 and p18 less than 5% of the gene expression. These findings suggest that the prevalence of cell cycle inhibition is caused by the upregulation of p21/CDKN1A. However, since the observed accumulation of cells in the G0/G1 phase is only partial, additional inhibitory mechanisms might come into effect. Unexpectedly, overexpression of H19 had no effect on proliferation or formation of capillary-like structures. These results indicate that H19 is necessary, but not sufficient for proliferation and tubulogenesis processes in human EC. A possible reason might be an underlying complex molecular interplay, where the removal of one component leads to its interruption. Conversely, the overexpression of one element might remain without effect, if one or more additional components are not overexpressed as well.

Since evidence was brought that H19 is mostly expressed in the endothelium of human muscle, the effects of H19 identified here in primary cell lines might be relevant also in a tissue context.

For H19, multiple mechanisms of action influencing proliferation have been described mainly in cancer cells, e.g. acting as sponge or as reservoir for miRNAs and thus modulating their target genes, or binding directly to a miRNA or a protein^44^. It is tempting to speculate that, at least in part, these mechanisms might be relevant also in ECs. Nuclear H19 binds EZH2, a key component of Polycomb repressive complex 2, and inhibits the transcription of a selective group of genes, thereby promoting bladder cancer metastasis^42^. MicroRNA-675, encoded in the first exon of H19, regulates cell proliferation and migration through CDK6, a pivotal regulator in cell cycle, in glioma and predicting a poor prognosis of glioma patients^46^. However, this miRNA was not detectable in hypoxic HUVEC^47^. H19 can promote metastasis through sponging and sequestering of let-7 miRNA^44^ and functions also as a “competing endogenous RNA” (ceRNA) for let-7 to control muscle differentiation^48^. Since let-7 has also been shown to be highly induced by hypoxia^49^, this interaction might be particularly interesting for elucidating the mechanisms underpinning HUVEC proliferation by H19. In accordance with its negative role in muscle differentiation, it has been recently reported that H19 may act as a scaffold that favors KSRP-mediated degradation of myogenin and other labile transcripts^50^,^51^. Indeed, H19 likely acts as molecular scaffold that favors KSRP-mediated degradation^50^. However, none of the KSRP target transcripts was modulated in hypoxic HUVEC, suggesting that H19 acts in a KSRP-independent way in this context.

In conclusion, next-generation sequencing approach was used to identify a high-confidence transcriptional signature, describing protein-coding and long non-coding RNAs modulated in human ECs. The present study may contribute to provide further insight into the biological functions and molecular mechanisms of lncRNAs related to diseases where hypoxia plays a crucial role, like myocardial ischemia, stroke or tumorigenesis.

## Methods

### Cell Cultures

HUVEC were cultured under normoxic and hypoxic conditions (1% oxygen) as described before^47^. HAOEC (ECACC) were grown in EGM-2 medium (Lonza) containing 2% FBS. The 293FT cell line (Thermo Fisher Scientific Inc.) was amplified in Dulbecco’s modified Eagle’s medium (DMEM), containing 10% fetal bovine serum and 500 μg/mL Geneticin (GIBCO). NIH 3T3 mouse fibroblast cell line were cultured in DMEM containing 10% fetal bovine serum. Phoenix Ampho (ATCC) cells were cultured as previously described^52^.

### Hindlimb ischemia model

Unilateral hindlimb ischemia was induced by femoral artery dissection in 2–3 months old male C57BL6 mice as previously described^18^. All experimental procedures were carried out in accordance with the Guidelines of the Italian National Institutes of Health and with the Guide for the Care and Use of Laboratory Animals (Institute of Laboratory Animal Resources, National Academy of Sciences, Bethesda, Md), were approved by the Institutional Animal Care and Use Committee of San Raffaele Scientific Institute, Milan, Italy and were authorized by “Ministero della Salute” (IACUC n. 666).

### Total RNA Isolation

*TRIzol* reagent was used to isolate total RNA from cells or tissues following the manufacturer’s instructions (Thermo Fisher Scientific Inc.). The purity and integrity of the obtained RNAs was measured by Nanodrop (Thermo Fisher Scientific Inc.) and Bioanalyzer (Agilent Technologies).

### H19 knockdown and overexpression

H19 was overexpressed using lentiviral vectors. Viruses were produced by transfecting 70% confluent 293FT cells with lentiviral vectors (pReceiver-Lv105, Genecopeia Inc) expressing human H19 or Empty Vector Control, using the the FuGENE 6 Transfection Reagent protocol (Promega). HUVEC cells were infected at 24 h and 48 h after plating with lentiviral supernatant, adding polybrene (8 ng/mL) (Sigma). 24 h after the last infection, cell were selected with puromycine 1 ug/ml.

For H19 knockdown, 50 nM of antisense LNA™ GapmeRs customer-designed for H19 or Negative control A (Exiqon) were transfected by siRNA transfection reagent (Santa Cruz Biotechnology) in 40% confluent HUVECs or HAOEC according to the manufacturer’s manual. For growth curve experiments, transfected HUVEC were seeded after 24 h, for matrigel-assays, after 48 h.

### Growth curves and capillary-like network formation

For growth curves, trypan blue negative cells were measured by the Countess cell counter (Thermo Fisher Scientific Inc.).

For capillary-like network formation, transfected HUVEC were seeded at a density of 18.000 on 96-well plates coated with matrigel (Cultrex Reduced Growth Factor Basement Membrane Extract, PathClear). Next, the number of branches of each node was counted.

### Cell cycle analysis

HUVEC were processed after 48 h of transfection with Click-iT EdU Alexa Fluor 647 Flow Cytometry Assay Kit (Thermo Fisher Scientific Inc.), according to the manufacturer’s protocol. Cells were then stained with propidium iodide and cell cycle analysis was performed on a Navios flow cytometer (Beckman Coulter) and analysed using Kaluza software (v1.2, Beckman Coulter).

### Apoptosis Assay

After 48 h of transfection, apoptosis was assessed in HUVEC using the Cell Death Detection Elisa (Roche) according to manufacturer’s instructions. Absorbance was measured on the multilabel counter Victor3 (Perkin Elmer).

### RNA-Sequencing and Preprocessing

For RNA-sequencing, total RNAs of HUVEC from two independent experiments with different time-points of hypoxia exposure (24 and 48 hours) and normoxia were extracted. The TruSeq RNA Sample preparation kit (Illumina) was used to generate poly-A selected, indexed cDNA libraries, according to the manufacturer’s instructions. Two libraries per lane were pooled and loaded onto a Illumina v3 Flow cell. Paired-end sequencing with a read length of 2 × 100 bp was performed separately for each experiment, using the Illumina HiSeq 2000 platform according to Illumina protocols. Quality control of the obtained reads was performed using FastQC suite with default parameters (http://www.bioinformatics.babraham.ac.uk/projects/fastqc/).

### Differential Gene expression in hypoxic HUVEC

For transcriptomic analyses, quality-score filtered sequences were aligned to the human genome assembly of Ensembl (GRCh37/ hg19, Feb 2009, Version 72) using the SoapSplice alignment software^53^. The number of uniquely mapping reads was estimated for each sample by using HTSeq-Count^54^. Gene expression read counts were used to identify differential expressed genes (DEGs) with DESeq2^55^, removing first weakly expressed genes by independent filtering. Then, generalized linear models are applied to identify differential expression. All hypoxic samples were compared to all normoxic samples, taking the two different sequencing runs into account.

Only lncRNAs unambiguously assigned by Ensembl to the transcript type “Long noncoding” were considered.

### Transcriptomic analysis by microarrays

The Ambion Illumina RNA amplification kit (Life Technologies) was used to process total RNAs. The obtained cRNAs were hybridized on a HumanHT-12 v4 Expression BeadChip, scanned and the background subtracted intensities were generated by GenomeStudio Software. Between arrays normalization (quantile) and differential expression was performed by limma package (Bioconductor, RStudio v 0.98.501,).

### Enrichment analysis

The hypoxic signature of HUVEC obtained by RNA-sequencing was separated in up- and down- modulated genes. Enrichr tool^56^ was used to perform enrichment analysis with gene ontology biological processes (GOBP) and transcription factors (ChIP Enrichment Analysis, ChEA).

The transcriptomic signature of HAOEC with inhibited H19 expression was investigated by WebGestalt^23^ for significant enrichment associated with disease terms, using all genes with detection p-value < 0.01 as reference set.

### Comparison of deep-sequencing with microarrays

The transcriptomic expression profile of hypoxic HUVEC measured by microarray analysis was intersected with the signature identified by RNA-sequencing. Log2 fold changes were correlated by GraphPad Prism software (version 4.00), testing first for normal-distribution and consequently performing non-parametric Spearman correlation.

### Real-time reverse transcriptase qPCR

For validation, isolated total RNA was first retro-transcribed using the SuperScript III Reverse Transcriptase kit and then investigated by SYBR green qPCR, according to the manufacturer’s protocol (Thermo Fisher Scientific Inc.). Primer couples were designed by Primer-BLAST tool. Cyclin/CDK inhibitors (p21/CDKN1A, p27/CDKN1B, p57/CDKN1C, p15/CDKN2B, p16/CDKN2A, p18/CDKN2C, p19/CDKN2D) were assayed with the TaqMan gene expression systems following the manufacturer’s recommendations (Thermo Fisher Scientific Inc.). The relative expression was calculated using the comparative Ct method 2^−∆∆CT^ as described previously^47^, normalizing to the averaged raw Cts of the RPL13 and/or UBC.

### Mouse miR-210 host gene

For detection of a potential novel transcript in the region of miR-210, publicly available RNA-sequencing data of differentiated 3T3-L1 cells in normoxia and hypoxia (1% oxygen for 24 h) were used (GSE35724)^19^. The data set was processed by Spliced Transcripts Alignment to a Reference (STAR) software^57^, followed by reference annotation based transcript assembly analysis (RABT) with the cufflinks software^58^, giving as -g option the mm10 Ensembl transcriptome.

### Identification of HIF binding sites

The regulatory regions (5 kbp upstream) and gene bodies of signature-lncRNAs were investigated taking advantage of a publicly available ChIP-Seq analysis of HIF binding sites identified in MCF-7 breast cancer cells after 16 hours of 0.5% ambient oxygen (GSE283521)^20^. MACS v2^59^ was used for peak calling with FDR < 0.01. The Position Weight Matrix (PWM) for the Hypoxia Responsive Element (HRE) was obtained from the MotifDb R package, while HRE hits were identified with the Biostring R package setting a minimum score of 80% and visualized with the Gviz R package.

### RNA *in situ* hybridization (ISH) assay and immunohistochemistry

Human muscle biopsies from biceps brachii were snap-frozen in cooled isopentane. All biopsy specimens were taken after informed consent disclosing future use for research was obtained. This study was authorized by the Institutional Ethics Committee, and was conducted according to the institutional regulation and Italian laws and guidelines. Muscle biopsies were sectioned (6 μm) and mounted on Superfrost Plus Gold glass slides (Thermo Fisher Scientific) dried for 30 min at room temperature and fixed in 2% NBF for 30 min at 4 °C. ISH experiments were performed using QuantiGene ViewRNA (Affymetrix) according to manufacturer’s instructions. Slides were labelled with H19 oligonucleotide probes conjugated with alkaline phosphatase type 1 followed by the addition of fast blue substrate. The specificity of H19 signal was confirmed by negative controls performed using a probe for DapB of *B. subtilis*. Finally, the slides were counterstained with Hoechst 3332 (Sigma-Aldrich Co.) and mounted with VectaMount AQ medium (Vector Lab., USA). Random images were acquired using the Axio Imager M.1 microscope equipped with an Axiocam MRc5 camera (Zeiss) and AxioVision software (Zeiss). To identify EC, serial muscle sections were incubated with anti-Human CD31 mouse monoclonal antibody (M0823 Dako). After incubation with biotinylated secondary antibody, and with ABC complex (Vectastain), the reaction was revealed with diaminobenzidine (DAB, Vector) and counterstained with Hematoxilin.

### Statistical Analysis

The two-tailed Student’s t test was used to analyse differences between normoxic and hypoxic conditions and a p < 0.05 was deemed statistically significant. Values are expressed as ±standard error.

## Additional Information

**Accession codes:** Datasets are deposited in Gene Expression Omnibus (GEO) database (RNA-sequencing of hypoxic HUVEC: **GSE76743**; microarray of H19 knockdown: **GSE76741**; microarray of hypoxic HUVEC: **GSE76739**).

**How to cite this article**: Voellenkle, C. *et al*. Implication of Long noncoding RNAs in the endothelial cell response to hypoxia revealed by RNA-sequencing. *Sci. Rep.* **6**, 24141; doi: 10.1038/srep24141 (2016).

## Supplementary Material

Supplementary Figures and Tables

Supplementary Tables S3_S4_S6

## Figures and Tables

**Table 1:** Validation of differentially expressed lncRNAs identified by RNA-sequencing in hypoxic HUVEC.

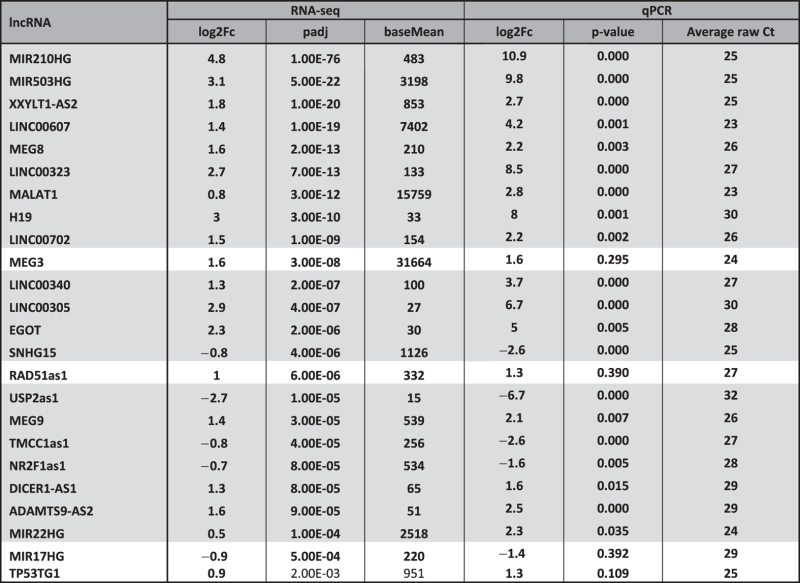

Details of all lncRNAs investigated conclusively by qPCR are shown. The lncRNAs confirmed in dependent and independent samples are highlighted in grey (20 out of 24, n = 8). Description of headers: “baseMean”=mean of normalized counts for all samples; “log2Fc”=log2 fold change (hypoxia versus normoxia). Table is sorted by increasing Benjamini-Hochberg adjusted p-value (=”padj”).
