# Supplementary material for: Implication of Long noncoding RNAs in the endothelial cell response to hypoxia revealed by RNA-sequencing
Source: Sci Rep. 2016 Apr 11;6:24141. doi: 10.1038/srep24141 (PMC4827084; doi:10.1038/srep24141)
Supplement: Supplementary Figures and Tables [file srep24141-s1.pdf]

SUPPLEMENTARY INFORMATION

Implication of Long noncoding RNAs in the endothelial cell response to hypoxia revealed by RNA-sequencing.

Voellenkle C., Garcia-Manteiga J. M., Pedrotti S., Perfetti A., De Toma I., Da Silva D., Maimone B., Greco S.,

Fasanaro P., Creo P., Zaccagnini, G. , Gaetano C., Martelli F.

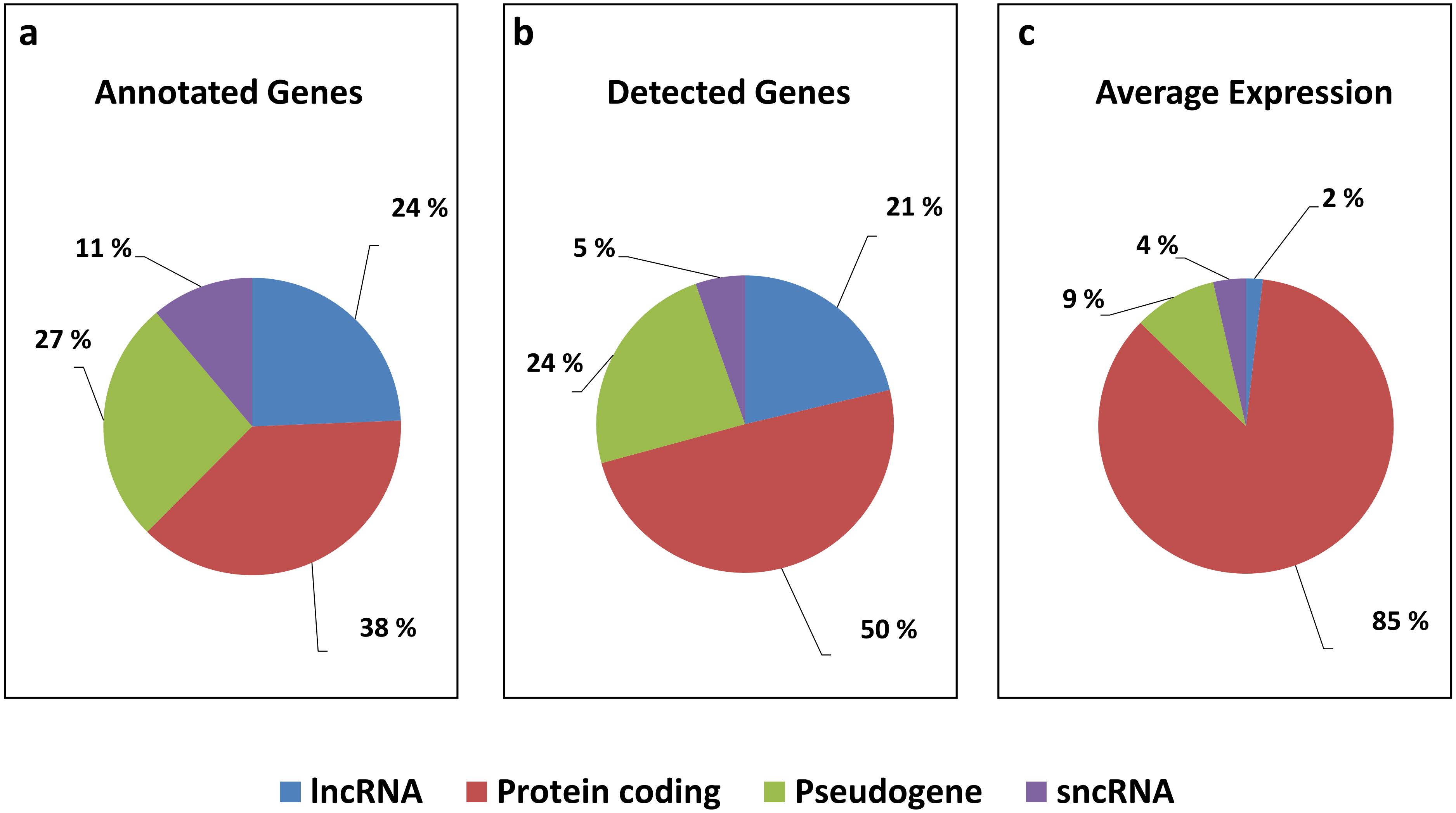

Supplementary Figure S1. Distribution of genes between classes and their percentage expression in HUVEC. Pie chart of the distribution of 4 gene classes as **(a)** annotated by Ensembl 72 and **(b)** detected by RNA-sequencing. **c)** Distribution of the average expression per gene between the 4 subclasses as measured by RNA-sequencing in HUVEC.

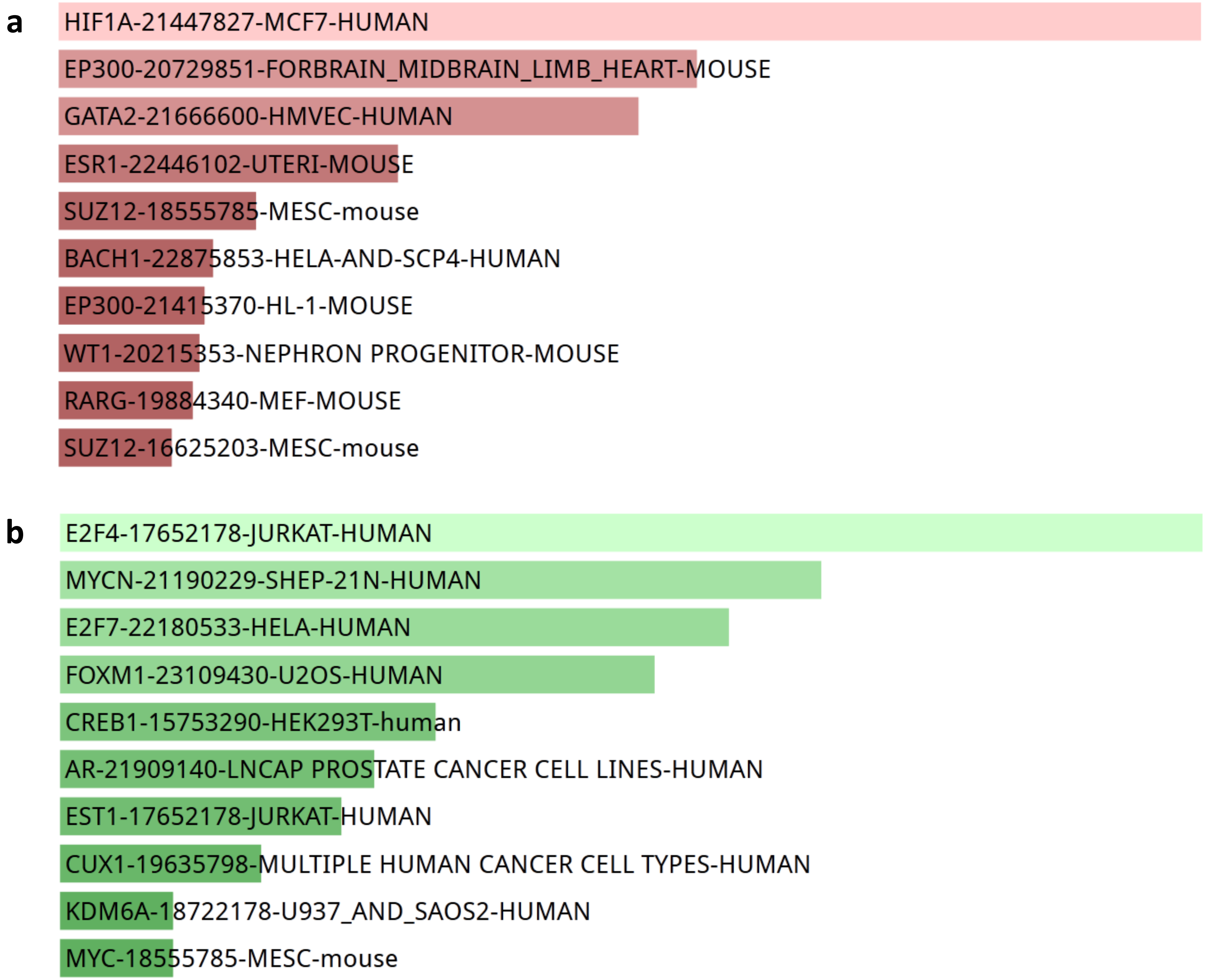

Supplementary Figure S2. Enrichment Analysis of transcription factor binding site profiles (ChEA) of genes modulated in HUVEC upon hypoxia exposure. Top 10 transcription factors associated by Enrichr analysis tool to **(a)** up-modulated and **(b)** down-modulated hypoxic signatures. Positions are ordered by rank based ranking. The Benjamini-Hochberg adjusted p-value <0.05 was used as significance threshold. The bar graph shows the transcription factor together with the PMID, cell type and species of the relevant study. The bars provide a visual representation of how significant each term is, the longer and lighter colored bars identifying more significant terms.

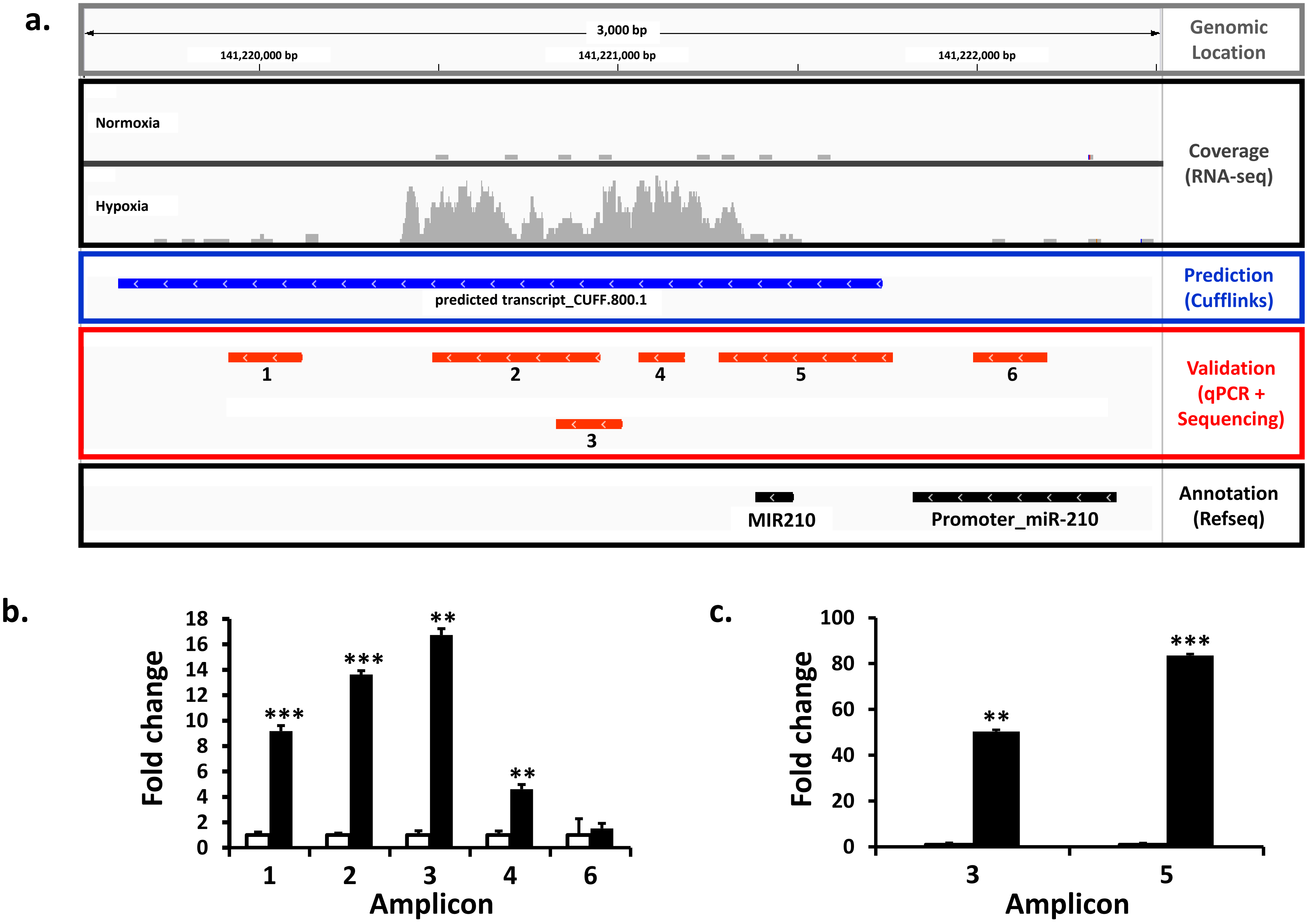

Supplementary Figure S3. Putative transcript of mouse miR-210 Host Gene. a) IGV view of miR-210 region displaying the genomic location (hg19), the coverage in hypoxic and normoxic samples of differentiated 3T3-L1 cells (GSE35724) as derived by RNA-seq. Blue track: transcript predicted by cufflinks suite. Red tracks: expressed amplicons identified by qPCR and confirmed by Sanger sequencing. Black track: annotated MIR210 and its promoter region. Bar graphs show the fold changes of **b)** identified amplicons measured in normoxic (white bar) and hypoxic (black bar) NIH3T3 mouse fibroblasts (n=3, **p<0.01, ***p<0.001) and **c)** identified amplicons at 7 days of ischemia, measured in a mouse model of hindlimb ischemia (white bar, normoperfused contralateral muscle, black bar, ischemic muscle; n= 3, **p<0.01, ***p<0.001).

a

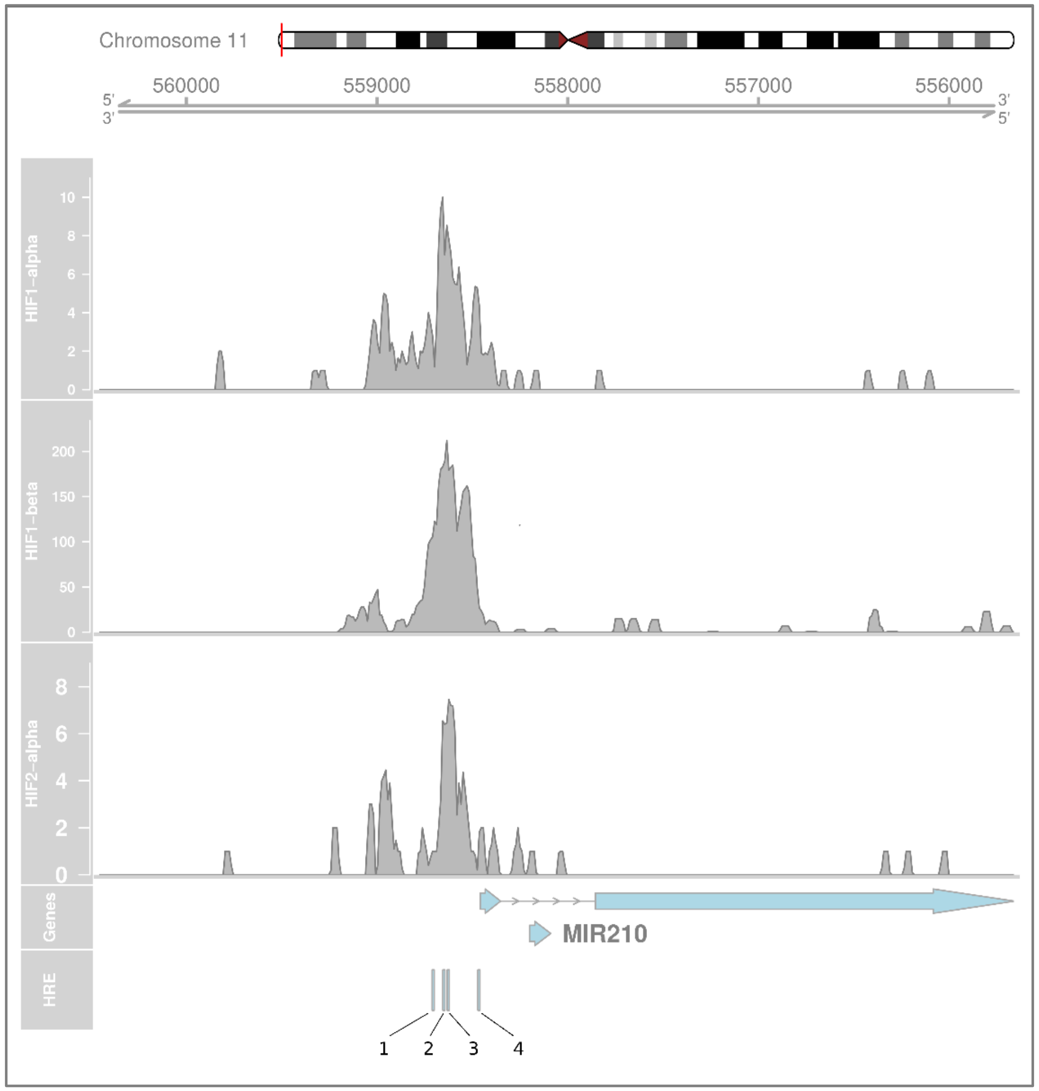

b

HRE Consensus Motif	HRE	Chr.	Start	End	Sequence	Score
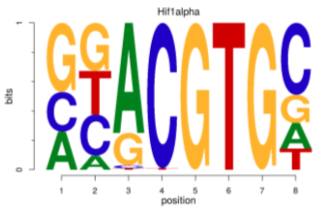	1	11	568694	568701	GGACGCGC	85
	2	11	568638	568645	GCAGGTGC	85
	3	11	568615	568622	AGACGTGC	95
	4	11	568454	568461	GGGCGTGG	90

Supplementary Figure S4. Conserved HIF-binding motifs identified in MIR210HG. a) Coverage plot of HIF1-alpha, HIF1-beta, and HIF2-alpha derived from ChIP-seq experiments in MCF-7 cultured in 0.5% oxygen for 16h (GSE28352). The region for MIR210HG region including 2 kbp upstream of the transcription start site is shown (hg 18). The gene model for MIR210HG and MIR210 loci is shown; the arrows indicate the direction of transcription. The four blue boxes indicate putative HREs in the peak. **b)** Conserved HIF-binding motif and details on the four identified HRE, like genomic location (lift-over to hg 19), sequence and the minimum conservation score.

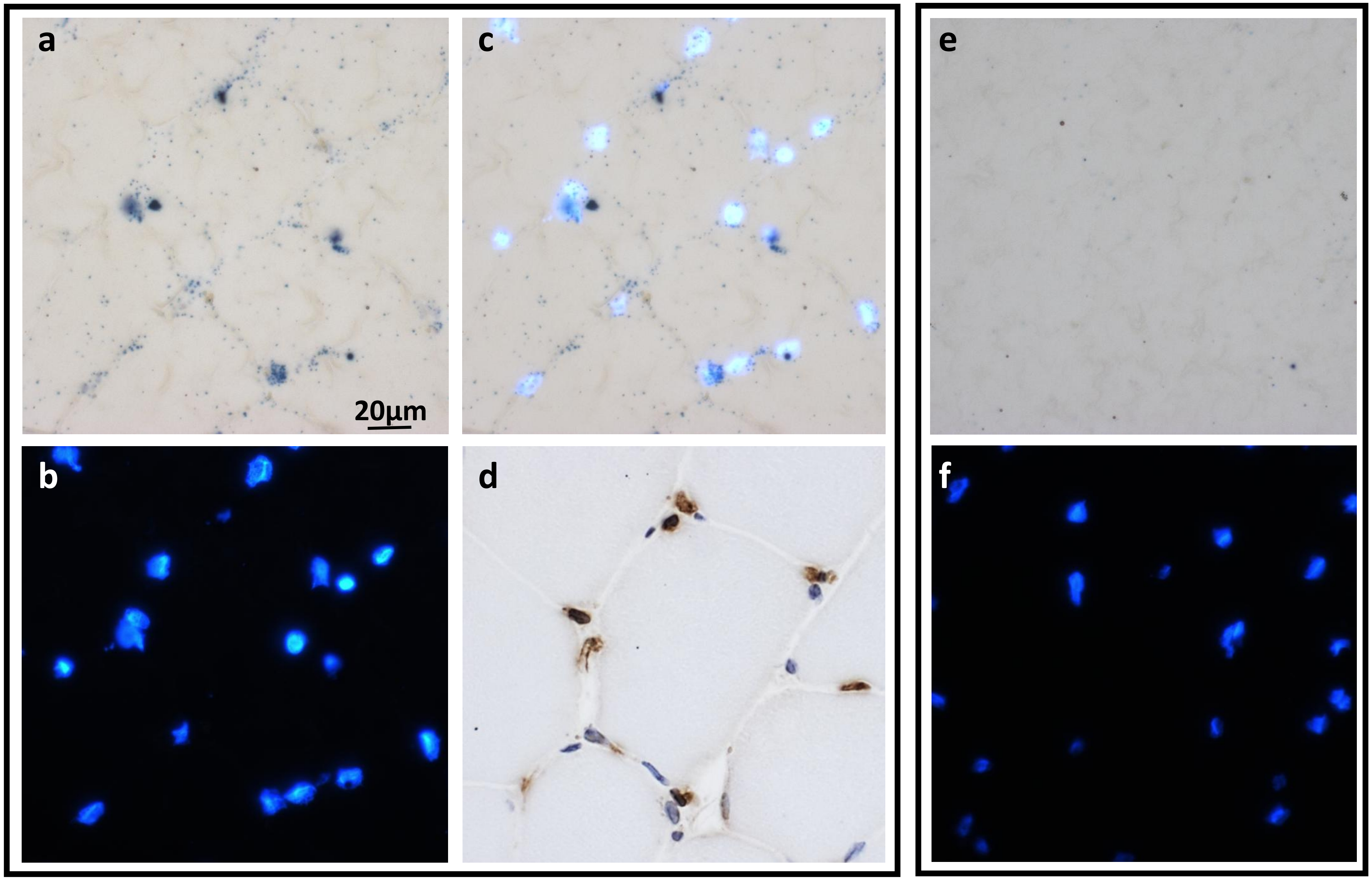

Supplementary Figure S5. H19 is expressed in blood vessels in the skeletal muscle. Serial sections were derived from frozen bopsies of human biceps brachii muscles. **H19- ISH. a)** Localization of H19 by using specific probes conjugated with alkaline phosphatase type 1 and fast blue substrate. **b)** Counterstaining with Hoechst 3332 **c)** Merge. **d) Endothelium- IHC.** Incubation of the serial section with anti-Human CD31 and biotinylated secondary antibody, followed by reaction of ABC complex with diaminobenzidine and counterstaining with Hematoxilin. **e) Negative control for ISH** performed using a probe for DapB of B. subtilis. **f)** Counterstaining with Hoechst 3332. H19 RNA is mostly expressed in the vascular tissue.

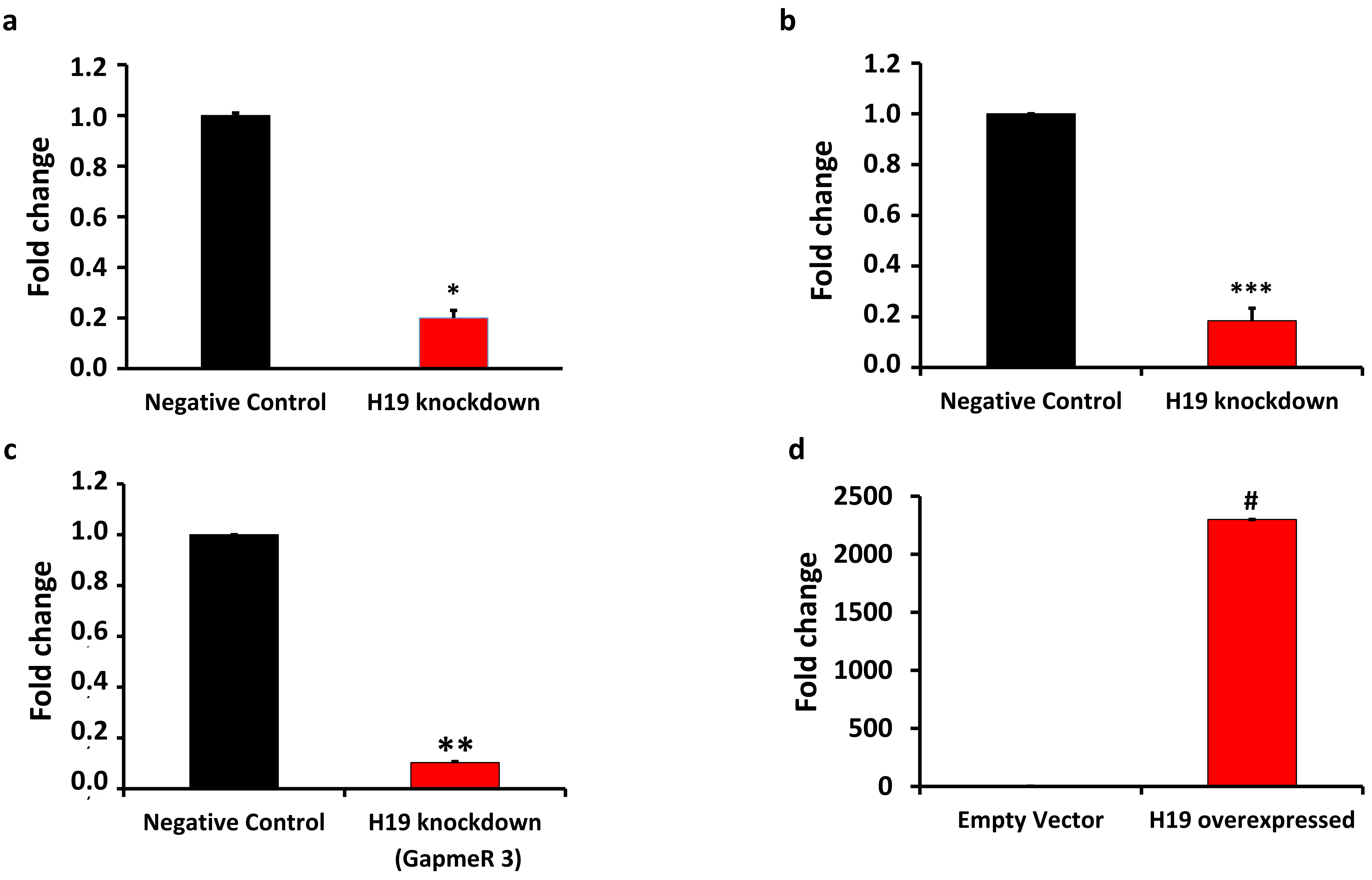

Supplementary Figure S6. Efficient H19 knockdown or overexpression in endothelial cells. a) H19 knockdown by transfection with an antisense oligonucleotide (LNA GapmeRs) in HAOEC (n=4; * p-value<0.05). **b)** H19 knockdown by transfection with an antisense oligonucleotide (LNA GapmeRs) in HUVEC (n=3; *** p-value< 0.001). **c)** H19 knockdown by transfection using a different LNA GapmeRs (oligo #3) in HUVEC (n= 3; ** p-value< 0.01). **d)** H19 overexpression by infection of HUVEC with lentiviruses expressing H19 (n= 3; # p<0.0001).

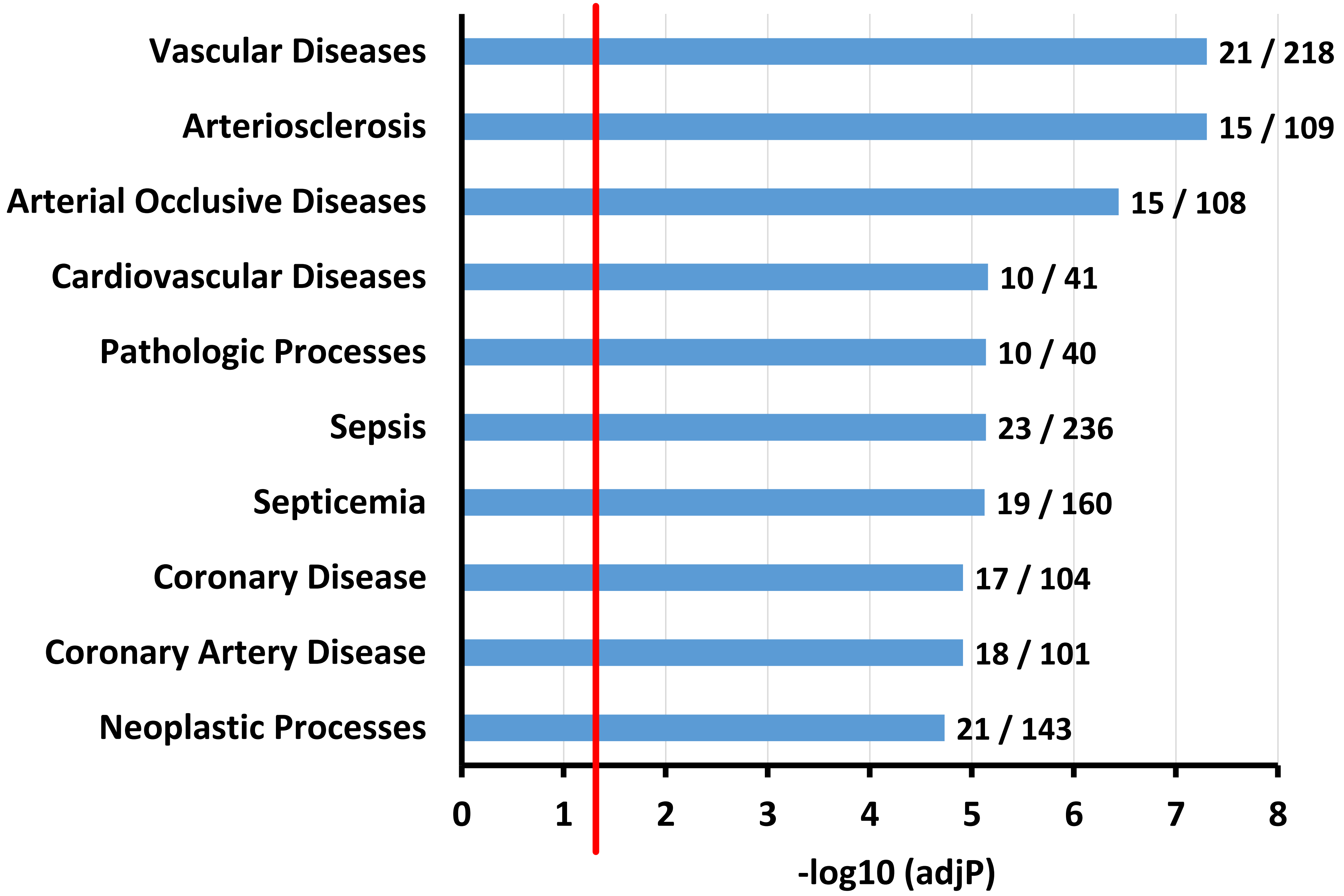

Supplementary Figure S7. Enrichment Analysis for genes modulated by H19 knockdown in HAOEC. WebGestalt tool was used to identify significant association between diseases and differentially expressed genes modulated by H19 knockdown in HAOEC. Top 10 diseases ranked by Benjamini-Hochberg adjusted p-value are shown. Numbers beside the bars indicate number of genes in common between H19 knockdown signature and the category, together with the number of all reference genes in the category. Significance threshold was set at adjusted p<0.05, shown as red line.

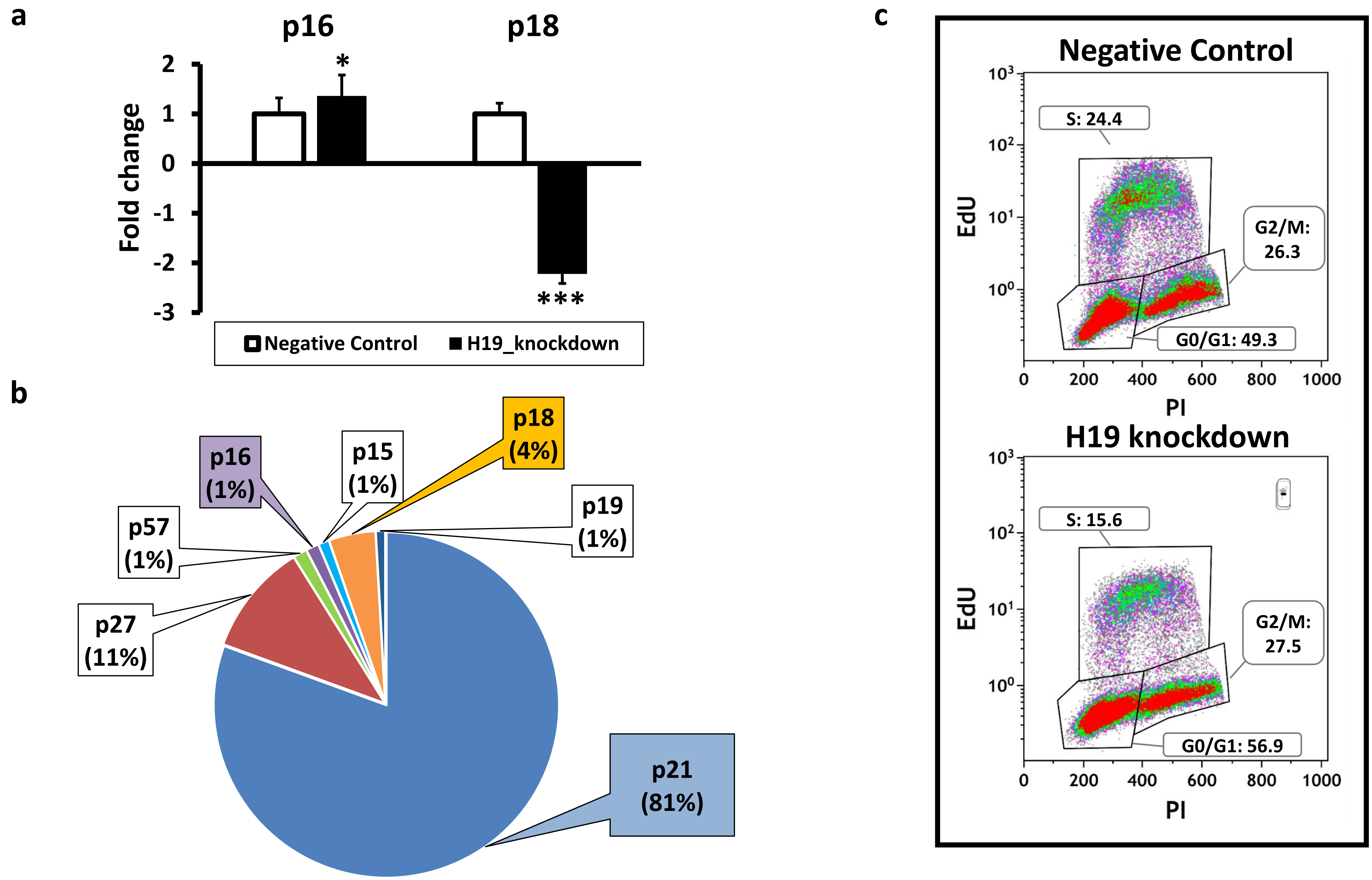

Supplementary Figure S8. Impact of H19 knockdown on the cell cycle. HUVEC were transfected with an antisense LNA GapmeRs specific for H19 or with negative control **a)** 48 hrs after transfection, total RNA was extracted and the expression of the indicated genes was assayed by qPCR. Significantly differential expressed cell cycle inhibitors identified by qPCR (n=8; *p<0.05 ***p<0.001). **b)** Distribution of expression levels among tested cyclin/CDK inhibitors as identified by RNA-sequencing in normoxic and hypoxic HUVEC. **c)** 48 hours after transfection with H19 antisense oligonucleotide or negative control, HUVEC were first labelled with EdU, then fixed and permeabilized. Incorporated EdU was revealed with an Alexa Fluor 647-based reagent, and total DNA content with propidium iodide. (n= 6). Representative ell cycle profiles obtained by FACS are shown, indicating a reduction of cells in S-phase and a proportional increase of cells in G0/G1.

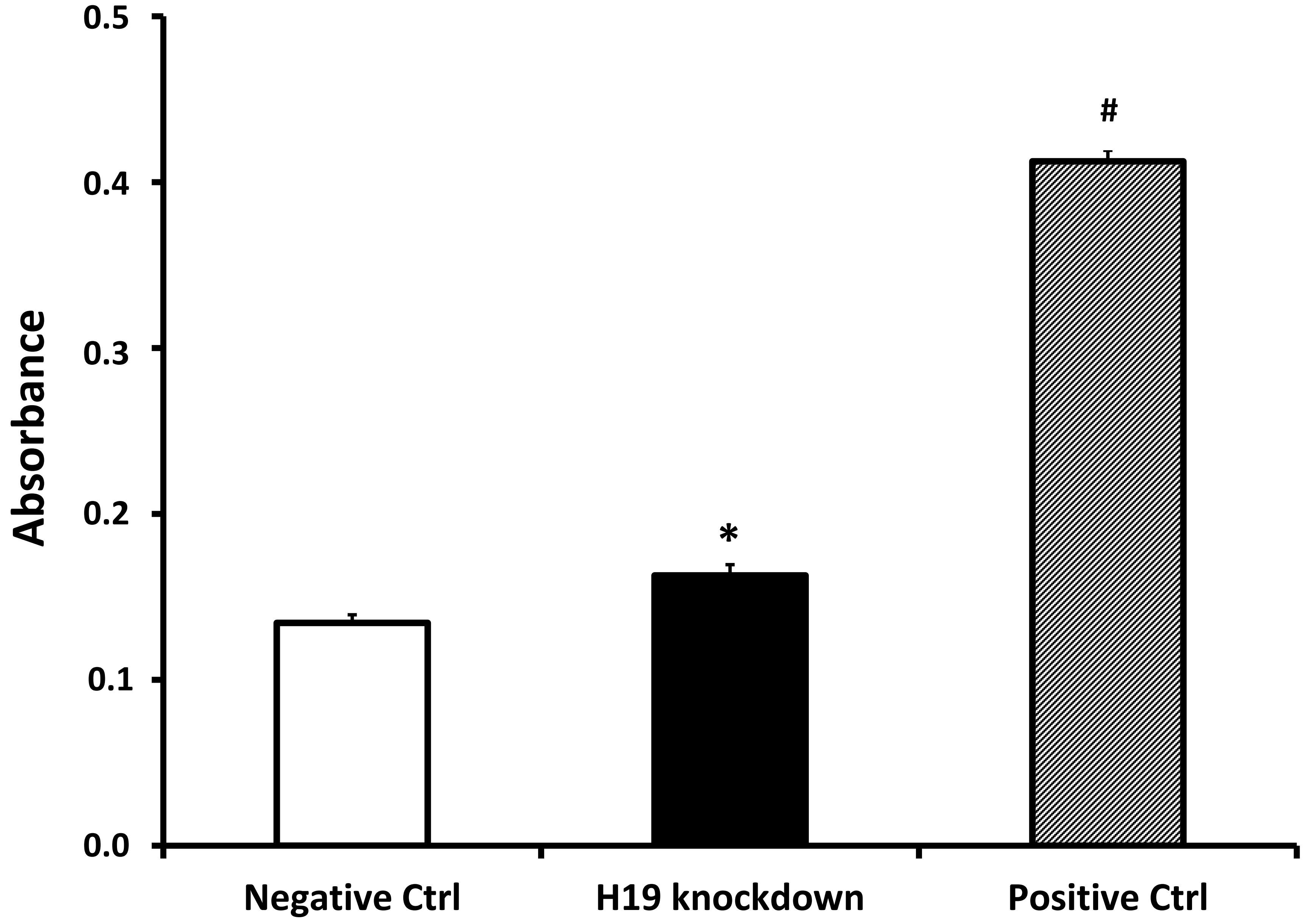

Supplementary Figure S9. Investigation of Apoptosis following H19 knockdown in HUVEC. 48 hours after transfection with H19 antisense LNA GapmeRs oligonucleotide or negative control, apoptosis was determined by quantifying fragmentation of cellular DNA by ELISA assay. Positive control is represented by HUVEC treated with 600uM H2O2 for 12 hours (n=3, *$p<0.05$, $^{\#}p<0.0001$).

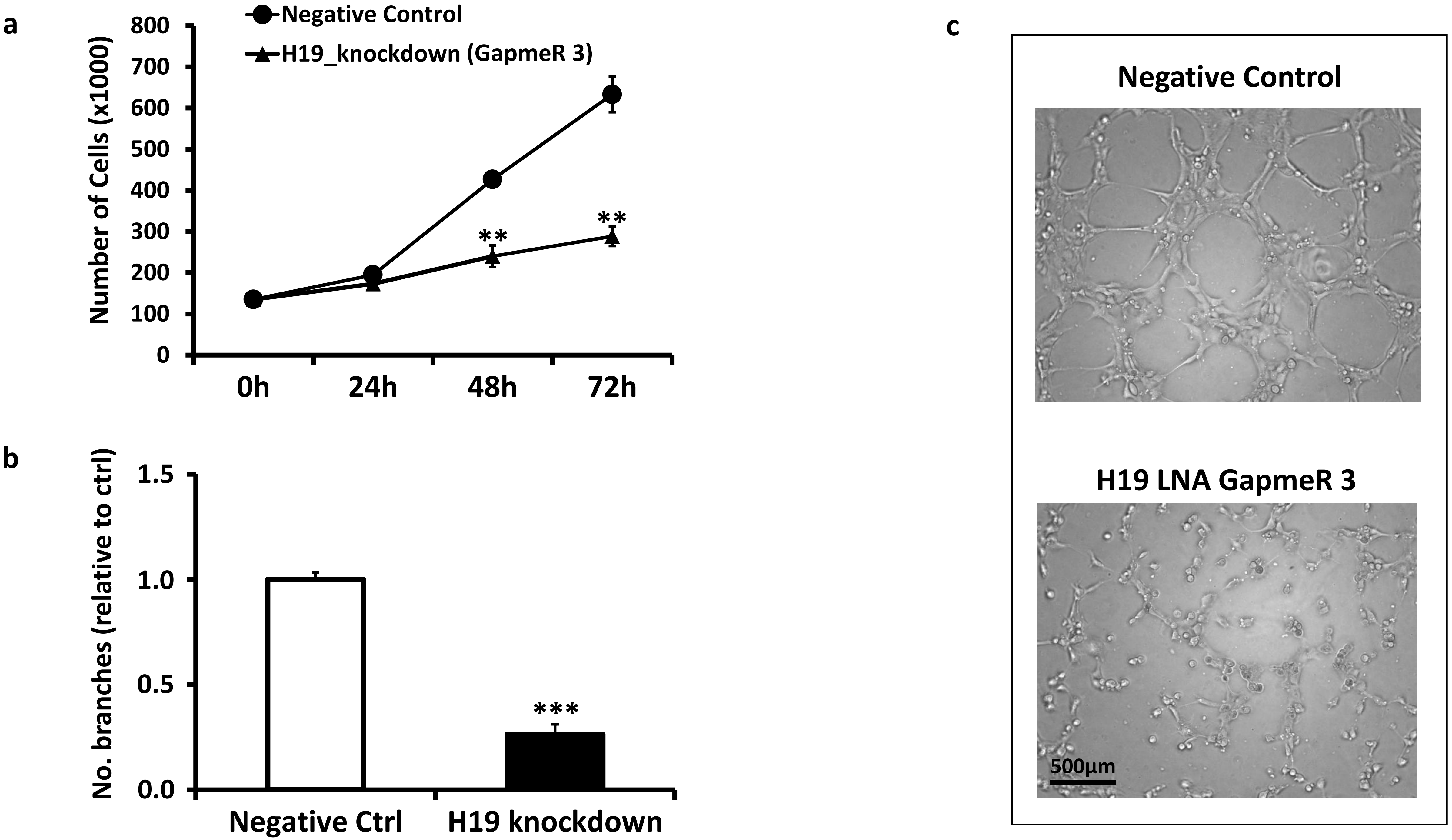

Supplementary Figure S10. Inhibition of proliferation and capillary-like structure formation upon H19 inhibition with an independent antisense LNA-GapmeR. HUVEC were transfected with a different antisense oligonucleotide (LNA GapmeR 3) specific for H19 or negative control. **a)** Proliferation assays were performed 24h after transfection. Growth curves show a significant impact of H19 knockdown compared to control (n=3, **p<0.01). **b)** The ability to form capillary like structures was quantified by matrigel-assay 48h after transfection, revealing a significant reduction upon H19 knockdown versus control (n=3, ***p<0.001). **c)** Representative phase-contrast images of the organization into capillary-like structures are shown**.**

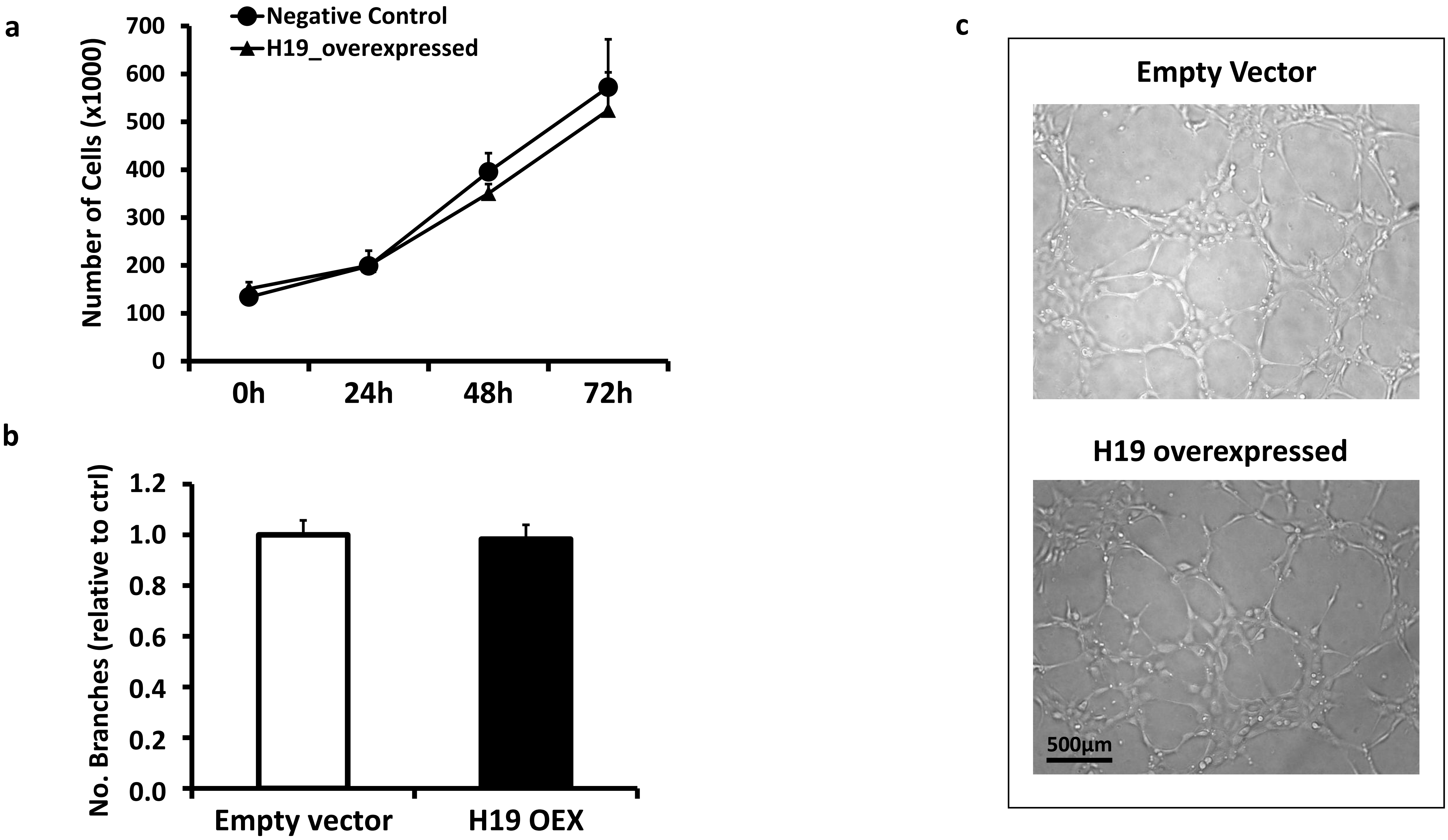

Supplementary Figure S11. H19 overexpression does not affect HUVEC proliferation and capillary-like structure formation. HUVEC were stably infected with lentiviruses expressing H19 or empty vector. **a)** Cell numbers were determined at different time points and growth curves were plotted (n=9). No significant differences in proliferation could be observed. **b)** Ability to form capillary like structures was analyzed by matrigel-assays. Quantitative assessment by counting the number of branches formed by HUVEC overexpressing H19 (H19 OEX) or empty vector revealed no significant difference (n=3)**. c)** Representative phase-contrast images of the organization into capillary-like structures are shown**.**

Supplementary Table S1: Next-Generation Sequencing Statistics. HUVEC samples exposed to Hypoxia (24 h or 48 h) or Normoxia (24 h) were sequenced. Quality control of sequenced reads was performed using FastQCsuite with default parameters. Fragments constituted by correct pairing of forward and reverse reads (R1 and R2) and aligning to exons of Ensembl (GRCh37/ hg19, Feb2009, Version 72) were estimated by SoapSplice alignment and HTseq-count software.

SAMPLE	SEQUENCED READS (R1+R2)	QC AND UNIQUELY ALIGNED READS	QC ALIGNED [%]	ALIGNED PAIRED	ALIGNED UNPAIRED	PROPERLY PAIRED [%]	FRAGMENTS MAPPED TO ANNOTATED EXONS
**Normoxia. 24 h**	164,263,996	144,483,110	88	135,242,633	9,240,477.00	94	62,838,282
**Normoxia. 24 h**	157,789,350	138,922,126	88	130,249,130	8,672,996.00	94	60,118,251
**Hypoxia. 24 h**	249,734,152	214,836,662	86	196,717,549	18,119,113.00	92	96,023,577
**Hypoxia. 24 h**	160,244,608	139,349,156	87	128,407,929	10,941,227.00	92	61,591,751
**Normoxia. 24 h**	286,029,390	246,665,524	86	226,088,573	20,576,951.00	92	111,773,694
**Normoxia. 24 h**	128,896,778	111,758,044	87	102,721,333	9,036,711.00	92	49,306,518
**Hypoxia. 48 h**	150,136,066	130,137,549	87	121,597,388	8,540,161.00	93	56,157,219
**Hypoxia. 48 h**	145,036,194	126,759,796	87	119,014,524	7,745,272.00	94	52,307,925

Supplementary Table S2. Genes and lncRNAs detected in normoxic and hypoxic HUVEC by RNA-sequencing. Numbers of all genes and the comprised lncRNA fraction as annotated in Ensembl version 72 compared to number of transcripts detected by HTSeq-Count at different expression levels. Differentially expressed (DE) genes and lncRNAs genes were identified by DESeq2.

	Genes	lncRNA
**Ensembl 72 (GRCh37/ hg19)**	**54,668**	**13,300**
**detected**<br>**(> 0 mean normalized counts)**	**34,773**	**7,381**
**considered for DE**<br>**(≥ 9.0 mean normalized counts)**	**19,596**	**2,458**
**DE (ctrl vs. Hypoxia)**<br>**(adjusted P <5e-5)**	**2,055**	**122**

Supplementary Table S5. Investigation of HIF transcription factor occupancy of lncRNAs differentially expressed upon Hypoxia. For this purpose, publicly available ChIP-seq data for HIF1-alpha, HIF1-beta, and HIF2-beta on hypoxic breast cancer MCF-7 cells were used. lncRNAs displaying at least one significant peak for one of the HIF proteins within the region of gene body and promoter (5 kbp upstream the TSS) are shown. The number of identified HREs are indicated for different conservation score levels (expressed in percentage). The fold change for each lncRNA as identified by RNA-sequencing is shown, up-regulation is highlighted in red, down-regulation in green. lncRNA validated by qPCR are highlighted in grey.

GeneName	HRE (80)	HRE (85)	HRE (90)	HRE (95)	log2FC
MIR210HG	4	2	1	1	4.8
RP11-77P16.4	0	0	0	0	3.3
CTC-378H22.1	1	0	0	0	3.0
H19	2	1	1	0	3.0
RP11-344E13.3	0	0	0	0	2.9
AP001046.5	0	0	0	0	2.8
AC010136.2	0	0	0	0	2.7
RP11-354K1.1	0	0	0	0	2.7
LINC00323	0	0	0	0	2.7
RP11-347E10.1	0	0	0	0	2.4
SLC2A1-AS1	4	2	1	1	2.2
ADORA2A-AS1	0	0	0	0	2.0
HIF1A-AS2	3	0	0	0	1.9
AC013400.2	0	0	0	0	1.9
RP3-510D11.2	1	0	0	0	1.8
LINC00277	1	1	1	0	1.8
RP5-995J12.2	0	0	0	0	1.7
RP3-399L15.3	0	0	0	0	1.6
MEG8	0	0	0	0	1.6
RP11-540A21.2	4	3	2	1	1.6
MEG3	0	0	0	0	1.6
LINC00702	0	0	0	0	1.5
RP11-235E17.6	0	0	0	0	1.5
AC093818.1	2	2	2	1	1.5
OPLAH	1	1	0	0	1.5
LINC00607	2	1	0	0	1.4
Z97634.5	1	1	0	0	1.4
LINC00340	0	0	0	0	1.3
RP11-93L9.1	0	0	0	0	1.3
RP11-818F20.5	0	0	0	0	1.3
RP11-492E3.1	5	3	2	0	1.1
PSMG3-AS1	2	2	1	0	1.1
CTC-205M6.5	0	0	0	0	1.0
AC002480.3	0	0	0	0	1.0
TPT1-AS1	5	1	0	0	0.9
AP001053.11	1	1	1	0	0.9
MALAT1	1	1	1	0	0.8
AC156455.1	1	0	0	0	0.8
LINC00847	2	0	0	0	0.7
PCBP1-AS1	7	5	4	0	0.7
BAIAP2-AS1	0	0	0	0	0.7
TMCC1as1	3	0	0	0	-0.8
RP11-111M22.3	2	1	1	0	-0.9
KB-1732A1.1	1	0	0	0	-1.1
PRIM2	0	0	0	0	-1.4
AC005682.5	0	0	0	0	-1.4
BCYRN1	0	0	0	0	-1.6
RECQL4	3	2	2	1	-1.6
RP11-132A1.4	0	0	0	0	-2.3
UHRF1	1	1	1	1	-2.4
USP2as1	2	0	0	0	-2.7
